# Complete Genome Sequences of Measles Virus Genotype D8 Isolates from South Korea in 2016

**DOI:** 10.1128/MRA.00032-19

**Published:** 2019-07-18

**Authors:** You-Jin Kim, Hae Ji Kang, Su-Jin Kim, Hye Min Lee, Sung Soon Kim

**Affiliations:** a Center for Infectious Diseases Research, National Institute of Health, Korea Centers for Disease Control & Prevention, Cheongju, Chungbuk, South Korea; b Center for Laboratory Control of Infectious Diseases, Korea Centers for Disease Control & Prevention, Cheongju, Chungbuk, South Korea; KU Leuven

## Abstract

The complete genome sequences of three wild-type measles viruses (genotype D8) isolated from patients in South Korea were determined. These are the first reported complete genome sequences of measles viruses obtained from South Korea, and the availability of these sequences will improve our understanding of measles virus transmission and genetic diversity.

## ANNOUNCEMENT

Measles is a highly contagious disease caused by the measles virus (MeV; genus *Morbillivirus*, family *Paramyxoviridae*). Vaccination can prevent infection, and accelerated immunization programs using a safe and cost-effective vaccine have decreased the global rate of measles-related deaths (http://www.who.int/news-room/fact-sheets/detail/measles).

Genetic information is a key factor for classifying viruses as endemic or imported, improving our understanding of virus transmission pathways (1). Laboratory-based MeV surveillance has emphasized the importance of adequate genetic analysis to verify virus elimination in a region, specifically through demonstrating the absence of endemic cases. For genotyping, the WHO recommends analyzing a sequence of 450 nucleotides in the C-terminal region of the N gene (N-450) (2). However, comparing these short sequences is not sufficiently informative for distinguishing some transmission routes, so whole-genome sequencing data will be helpful for analyzing strains (3).

Several imported and import-related measles cases occurred sequentially in South Korea in 2016. Here, we report the full-length genome sequences of MeV genotype D8 isolates obtained using Vero-hSLAM cells (4) from throat swab specimens of three presumed import-related cases, including a 38-year-old male who works at the Gimpo International Airport with no international travel history or clear epidemiological linkage with known domestic measles cases. Furthermore, two cases (a 1-year-old female and a 30-year-old male) had a history of close contact with known imported cases from Indonesia, according to a national epidemiological investigation. Total RNA was extracted from cultivated viruses with an RNeasy minikit (Qiagen, CA, USA) and then reverse-transcribed using SMARTScribe reverse transcriptase (Clontech, CA, USA) and random hexamers. The viral genome was PCR amplified using gene-specific primers designed based on previously published MeV sequences (Table 1). Nucleotide sequences were directly sequenced in an ABI 3730XL DNA analyzer (GnC Bio Co., South Korea) and assembled using Sequencher version 3.1 (Insilicogen, South Korea). Then, 5′- and 3′-terminal end sequences were confirmed through rapid amplification of cDNA ends (RACE) (5). The complete genomes of the three MeV strains (MVs.Seoul.KOR.37.16[D8], MVs.Changwon.KOR.39.16.2[D8], and MVs.Geoje.KOR.41.16[D8]) each contained 15,894 nucleotides and showed highest identities (99.89%, 99.77%, and 99.89%, respectively) with the complete genome of MVs/Brisbane.AUS/33.15[D8]. Within the N-450 window, one to three nucleotide positions (0.22 to 0.66%) differed between the three strains. Additionally, analysis in Molecular Evolutionary Genetics Analysis (MEGA) version 6 revealed that the pairwise distances of the three complete genomes were 0.0018 to 0.0030 at the nucleotide level (6). The comparison of measles virus sequences from related known imported cases and these cases is limited due to a lack of genome sequences from imported cases.

**TABLE 1:** Gene-specific primers for sequencing of measles virus genome

Application^*a*^	Primer name	Direction	Sequence (from 5′ to 3′)	Length (bp)	Nucleotide region	Amplicon size (bp)
RT-PCR 1	Me-NF	Forward	GCCGCGGACCAAACAAAGTTGGGTAAGG	28	1–21	1,582
	Me-N-1R	Reverse	GCTGACCTTCGACTGTCCTG	20	1563–1582	
RT-PCR 2	Me-N-1L	Forward	CCAGACAAGCTCAAGTGTCG	20	1342–1360	1,327
	Me-PVC-1R	Reverse	CGGGTGTCCACTCCTGTATC	20	2652–2668	
RT-PCR 3	Me-PVC-1L	Forward	CTCATTTGGAACGGAGATCG	20	2517–2536	1,306
	Me-M-1R	Reverse	GGACCTTTCTCCAAGGTGTG	20	3803–3822	
RT-PCR 4	ME-M-1L	Forward	CTGGTCTAGGCGACAGGAAG	20	3556–3575	1,293
	ME-M-2R	Reverse	CTAGGGAGTCGGATGTCTGC	20	4819–4838	
RT-PCR 5	ME-M-2L	Forward	AAGACTCCACAGGCCAAGC	19	4526–4544	1,279
	ME-F-1R	Reverse	ACTCCCGCAAATCTCTTGTG	20	5785–5804	
RT-PCR 6	ME-F-1L	Forward	AACACCCACCGGTCAAATC	19	5514–5532	1,201
	ME-F-2R	Reverse	GTGATCGGCAGCAATGTATG	20	6697–6714	
RT-PCR 7	ME-F-2L	Forward	GTCCTCTGCTCCAAGAATGC	20	6512–6531	1,289
	ME-H-1R	Reverse	GTTGCCCTGGTCTCCAGTAG	20	7781–7800	
RT-PCR 8	ME-H-1L	Forward	GATGAAGTGGGCTTGAGGAC	20	7574–7590	1,173
	ME-H-2R	Reverse	CTCGCCTGCTTCCTTAATTG	20	8727–8746	
RT-PCR 9	ME-H-2L	Forward	CCATTGATCACACACGGTTC	20	8567–8586	1,154
	ME-L-2R	Reverse	CAAACCATTGGGAGCTATGC	20	9701–9720	
RT-PCR 10	Me-L-2L-2	Forward	AGGAGTTACCCGACCCACTC	20	9462–9481	1,203
	ME-L-3R	Reverse	ATCCCATTCCCTTTGGAGAG	20	10645–10664	
RT-PCR 11	ME-L-3L	Forward	CCCAGACTTGAAGCGGTAAC	20	10308–10327	1,189
	ME-L-4R	Reverse	ATAGGGAATGGTGCTGATGG	20	11476–11496	
RT-PCR 12	ME-L-4L	Forward	TTGCCCAGAGGCTAAATGAG	20	11271–11288	1,126
	ME-L-5R	Reverse	TGATATGCCTGTCCATGAGG	20	12377–12396	
RT-PCR 13	ME-L-5L	Forward	CTAATGCCTGAAGAGACCCT	20	12147–12166	1,008
	ME-L-6R	Reverse	CACTCGGACAAGAGATGTGC	20	13135–13154	
RT-PCR 14	ME-L-6L	Forward	AGACATGAAGCTTGCCTTCG	20	12896–12915	2,057
	ME-L-1R	Reverse	GGAGCAGAGCCATCGATAAG	20	14933–14952	
RT-PCR 15	Me-L-2L	Forward	TCTACTTAGGCCAGTGTGCAG	21	13687–13707	2,208
	Me-LR	Reverse	TTGGGTACCGACCAGACAAAGCTGGGAATAG	31	15874–15894	
RACE PCR 1	ME-NF-3	Forward	GGTACCTCTTGATGCGAAGG	20	451–470	
RACE PCR 2	ME-NF-2	Forward	GATCAATCCAGGTTCGGATG	20	525–544	
RACE PCR 3	Me-L-1L	Forward	AAGTTGGCCTTGTCGAACAC	20	14722–14741	
RACE PCR 4	ME-L-1L-2	Forward	GAAGCCAACAAGGGATGTTC	20	15457–15476	

a RT-PCR, reverse transcriptase PCR; RACE, rapid amplification of cDNA ends.

### Data availability.

The whole-genome sequences of the MVi/Seoul.KOR/37.16[D8], MVi/Geoje.KOR.41.16[D8], and MVi/Changwon.KOR.39.16/2[D8] isolates have been deposited in GenBank under the accession numbers MF496200, MF496201, and MF496202, respectively.

## References

[1] Rota PA, Bankamp B 2015 Whole-genome sequencing during measles outbreaks. J Infect Dis 212:1529–1530. doi:10.1093/infdis/jiv272. 26153410

[2] Bellini WJ, Rota PA 1998 Genetic diversity of wild-type measles viruses: implications for global measles elimination programs. Emerg Infect Dis 4:29–35. doi:10.3201/eid0401.980105. 9452396 PMC2627654

[3] Penedos AR, Myers R, Hadef B, Aladin F, Brown KE 2015 Assessment of the utility of whole genome sequencing of measles virus in the characterisation of outbreaks. PLoS One 10:e0143081. doi:10.1371/journal.pone.0143081. 26569100 PMC4646484

[4] WHO. 2018 Manual for the laboratory diagnosis of measles and rubella virus infection. World Health Organization, Geneva, Switzerland.

[5] Frohman MA 1994 On beyond classic RACE (rapid amplification of cDNA ends). PCR Methods Appl 4:S40–S58. doi:10.1101/gr.4.1.S40. 9018326

[6] Kumar S, Tamura K, Nei M 1994 MEGA: Molecular Evolutionary Genetics Analysis software for microcomputers. Comput Appl Biosci 10:189–191. 8019868 10.1093/bioinformatics/10.2.189

